# Urbanization disrupts latitude‐size rule in 17‐year cicadas

**DOI:** 10.1002/ece3.3879

**Published:** 2018-02-02

**Authors:** DeAnna E. Beasley, Clint A. Penick, Nana S. Boateng, Holly L. Menninger, Robert R. Dunn

**Affiliations:** ^1^ Department of Biology, Geology, and Environmental Science University of Tennessee at Chattanooga Chattanooga TN USA; ^2^ Department of Applied Ecology North Carolina State University Raleigh NC USA; ^3^ Keck Center for Behavioral Biology North Carolina State University Raleigh NC USA; ^4^ The Biomimicry Center Arizona State University Tempe AZ USA; ^5^ Bell Museum University of Minnesota St. Paul MN USA; ^6^ Center for Macroecology, Evolution and Climate Natural History Museum of Denmark University of Copenhagen Copenhagen Denmark

**Keywords:** Bergmann's rule, citizen science, fluctuating asymmetry, geometric morphometrics, periodical cicada, urban ecology

## Abstract

Many ectotherms show a decrease in body size with increasing latitude due to changes in climate, a pattern termed converse Bergmann's rule. Urban conditions—particularly warmer temperatures and fragmented landscapes—may impose stresses on development that could disrupt these body size patterns. To test the impact of urbanization on development and latitudinal trends in body size, we launched a citizen science project to collect periodical cicadas (*Magicicada septendecim*) from across their latitudinal range during the 2013 emergence of Brood II. Periodical cicadas are long‐lived insects whose distribution spans a broad latitudinal range covering both urban and rural habitats. We used a geometric morphometric approach to assess body size and developmental stress based on fluctuating asymmetry in wing shape. Body size of rural cicadas followed converse Bergmann's rule, but this pattern was disrupted in urban habitats. In the north, urban cicadas were larger than their rural counterparts, while southern populations showed little variation in body size between habitats. We detected no evidence of differences in developmental stress due to urbanization. To our knowledge, this is the first evidence that urbanization disrupts biogeographical trends in body size, and this pattern highlights how the effects of urbanization may differ over a species’ range.

## 1 INTRODUCTION

Urban habitats are characterized by increased temperatures and higher prevalence of environmental stressors that impact the biological processes of organisms relative to those living in rural environments (Grimm et al., 2008; Oke, 1973). Already there is evidence that urban conditions are shaping species’ traits on a local scale (Diamond & Martin, 2016; Donihue & Lambert, 2015), and the effects of urbanization will likely vary geographically from one end of a species range to the other. For example, urban heating at cooler extremes of a species’ range may be beneficial, while heating could have a negative impact on species in the warmer extremes of their range where populations may already be close to their physiological limits (Diamond, Frame, Martin, & Buckley, 2011; Diamond et al., 2013; Kingsolver, Diamond, & Buckley, 2013; Youngsteadt, Ernst, Dunn, & Frank, 2016). Likewise, organisms may be less tolerant of urban stressors (e.g., pollutants, fragmented landscapes, and changes in food resources) in parts of their range where they may be living close to their physiological limits. An understanding of how urbanization affects species across their range is therefore necessary to predict the impacts of urbanization on species with broad distributions.

Species with broad distributions often show predictable, clinal variation in body size and morphology (Blanckenhorn & Demont, 2004). Body size of endotherms tends to increase with increasing latitude while body size of ectotherms tends to decrease with increasing latitude. These two patterns are known, respectively, as Bergmann's rule and converse Bergmann's rule (Bergmann, 1848; Mousseau, 1997) and have been found to hold often, if not always (Blanckenhorn & Demont, 2004). In ectotherms, these patterns are likely due to direct effects of temperature and seasonal length on the fitness and physiology of individual organisms. This is particularly true for organisms that undergo molts, where warmer conditions may allow larvae and nymphs to grow larger before they molt and hence have a larger final size (Roff, 1980). Urban conditions, particularly urban heating, may disrupt ecological patterns in body size if they extend the growing season or change resource availability (Lowe, Wilder, & Hochuli, 2014).

While an organism's developmental pathway is expected to buffer itself against environmental disturbances (Markow, 1995), the compounded effects of urban stressors may compromise this ability (Hosken, Blanckenhorn, & Ward, 2000). Consequently, urban species may experience a breakdown in developmental stability that could cause a reduction in body size or increased levels of asymmetry, a sign of developmental stress. Higher levels of fluctuating asymmetry—a deviation from perfect, bilateral symmetry—would suggest fitness consequences that impact survival and mate selection (Møller & Thornhill, 1998; Parsons, 1990). In various ectotherms, fluctuating asymmetry has served as an indicator of environmental stress due to pollutants, radiation, malnutrition, or extreme temperatures (Beasley, Bonisoli‐Alquati, & Mousseau, 2013; Palmer & Strobeck, 2003). Thus, assessing changes in both body size and fluctuating asymmetry would provide further insight into how urban environments shape the evolutionary trajectories of these populations.

The aim of our study was to assess the effects of urbanization across the latitudinal range of periodical cicadas (*Magicicada spp*.). Periodical cicadas provide an ideal model for studying urban effects due to their long life cycle, synchronous emergence, and broad distribution across latitude and rural‐urban gradients (Williams, Smith, & Stephen, 1991). Previous work has demonstrated that periodical cicadas show clinal variation in body size consistent with converse Bergmann's rule, with smaller body size in cold, northern regions, and larger body size at the southern end of their range (Koyama et al., 2015). With the North American landscape projected to undergo significant changes due to urbanization (Terando et al., 2014) and the sensitivity of cicada development and activity to temperature and landscape structure (Heath, 1967; Karban, 2014; Moriyama & Numata, 2015), periodical cicadas may be particularly susceptible to the associated temperature and habitat changes (Cooley, Marshall, Simon, Neckermann, & Bunker, 2013; Gilbert & Klass, 2006).

To assess the impact of urban environments on cicada development, we launched a citizen science initiative to collect cicadas during the 2013 emergence of Brood II. Brood II is a population of single‐aged periodical cicadas that emerge on a 17‐year cycle in the eastern part of the United States with a range extending from Georgia in the south to Connecticut in the north (Simon, 1988). To determine whether urbanization increased signs of developmental stress, we quantified body size and fluctuating asymmetry in cicada wings. Because periodical cicadas have been found to follow converse Bergman's rule with smaller body size in cold, northern regions and because cities tend to be warmer than the surrounding area (Koyama et al., 2015; Oke, 1973), we expected to see an increase in cicada body size in cities compared with rural areas. Conversely, if cicadas at the southern end of their range are already living close to their physiological maximum, then we expected additional heat imposed by cities and other urban stressors to result in a reduction in body size and/or an increase in fluctuating asymmetry. By comparing rural and urban cicadas across their range, we evaluate how urbanization can have different impacts on development for cicadas and potentially other broadly distributed ectotherms.

## 2 MATERIALS AND METHODS

### 2.1 Study organism

**Figure 1:**
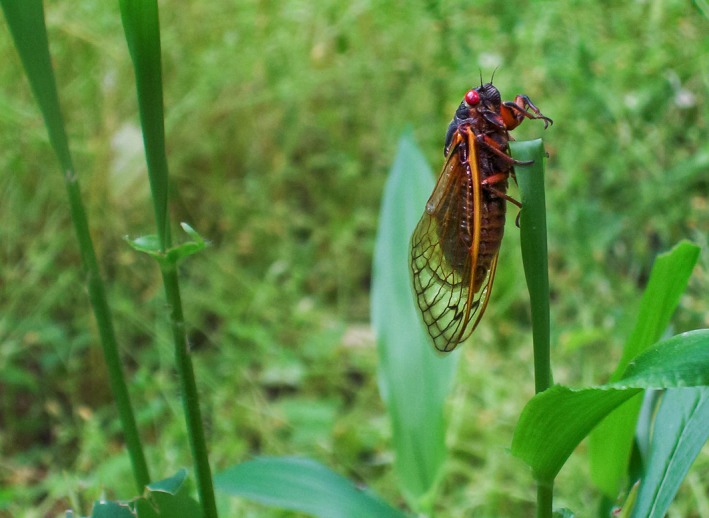
*Magicicada septendecim*

*Magicicada* is a genus of periodical cicadas that is found exclusively in the eastern half of North America and is made up of seven species (Williams & Simon, 1995). They spend most of their lives underground as nymphs, developing for either 13 years or 17 years depending on the species. All groups pass through five instars before molting into the adult form (White & Lloyd, 1975). During an emergence year, a single‐aged cohort of cicadas—known as a brood—emerge synchronously (Williams & Simon, 1995). The number of individuals in a single brood can range from 30,000 to 3.5 million per hectare. Adult periodical cicadas are characterized by having black bodies with orange‐veined wings and red eyes, easily distinguished from sympatric annual cicadas (Figure 1). They are most notable for their species‐specific mating calls, which in large aggregations can range from 50 to 80 decibels (Williams & Smith, 1991). Brood II is a 17‐year cohort found along the eastern coast of the United States that consists of three species (*M. septendecim, M. septendecula, M. cassini*) (Simon, 1988). Because its geographic range occurs in the most populated part of the United States, its emergence pattern is well‐known and covers a range of urban and rural environments (Dybas & Lloyd, 1974).

### 2.2 Sample collection

**Figure 2:**
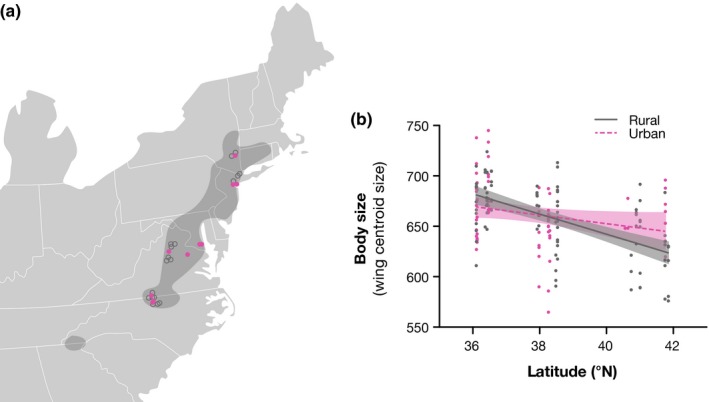
(a) Sampling locations during 2013 emergence mapped over the major range of Brood II. (b) Regression analysis shows periodical cicadas in rural locations (solid line) follow a converse Bergmann's rule with cicadas decreasing in size with increasing latitude. Urban cicadas in the northern part of the range (dashed line) do not follow the converse Bergmann's rule pattern

Periodical cicadas were collected during the 2013 Brood II emergence as part of the Urban Buzz: Periodical Cicada Citizen Science Project (http://robdunnlab.com/projects/urban-buzz/). Citizen scientists were recruited across the Brood II range and were instructed to collect 5‐10 dead, intact periodical cicadas from single locations and place them in a small plastic container with tissue paper or bubble wrap to ensure protection during transport. Each container was labeled with the citizen scientist's name, email address, specific collection location (i.e., address, GPS coordinates), and date. Upon arrival in the laboratory, individuals were sorted and stored in a ‐20 freezer until further analysis. For consistency, we only used *M. septendecim* specimens because they make up the majority of individuals in Brood II as well as in our collections (Leonard, 1964). *M. septendecim* was identified by the presence of broad orange stripes on the underside of the abdomen and orange coloration behind the eye (Leonard, 1964). Sex was identified by the presence (female) or absence (male) of the ovipositor. In total, citizen scientists collected 238 *M. septendecim* (of 272 total individuals) across five states from 29 independent locations during the emergence (Figure 2a). The sampling effort covered 88% of the latitudinal range of Brood II and 71% of *M. septendecim*'s overall latitudinal range.

### 2.3 Wing morphology and body size measurements

All samples were carefully assessed for damage that would prevent accurate measurements of wing structure. In total, 163 individuals were used for morphological analysis. Wings were removed from the body and laid flat on a clear mounting tray. Images were captured using a digital camera (PowerShot SX510 HS, Canon) mounted on a tripod. For shape asymmetry and size measurements, we selected 26 landmarks on wing vein intersections of the forewing (Klingenberg, Barluenga, & Meyer, 2002; Figure S1). Landmarks were digitized using TpsDig2.16 software (Rohlf, 2005). Because measurement precision is important for morphological analysis, we independently captured measurements three times to account for measurement error (Palmer & Strobeck, 2003). Measurements were taken in random order and blind to information on location and sex.

We used geometric morphometric techniques to test for measurement error, assess presence of other developmental asymmetries, and extract fluctuating asymmetry (FA) and wing size values using MorphoJ software (Klingenberg, 2011; Klingenberg & Monteiro, 2005). Landmarks from both the left and right wing were superimposed and rotated to achieve the overall best fit between corresponding landmarks by standardizing to a unit centroid size which we defined as an overall measure of wing size (Klingenberg & McIntyre, 1998). We then verified that wing centroid size was significantly correlated with thorax width, another standard metric used to estimate body size (*r*² = .24, *p* < .01). Thus, we used wing size as a proxy for overall body size of the individual, which is a common metric for quantifying insect body size (Gerard et al., 2015; Hoffmann, Collins, & Woods, 2002).

### 2.4 Urbanization gradient and climate

We derived latitude/longitude coordinates from submitted addresses for each sample location using Google Earth (https://www.google.com/earth/) and quantified urbanization by percentage impervious surface within 50 m using 2013 National Land Cover Database, NLCD (Fry et al., 2011). Impervious surface has been shown to be closely associated with urbanization factors, including land surface temperature and vegetation cover (Imhoff, Zhang, Wolfe, & Bounoua, 2010). A 50‐m radius around each sample location was selected to account for dispersal distance of periodical cicadas (Karban, 1981). Locations with less than 9% impervious surface were categorized as rural, while values 12% and greater were considered urban (McKinney, 2002). We obtained mean annual temperature (°C) and mean annual precipitation for each sample location using PRISM Climate Group dataset at a scale of 4 km grid cells (PRISM Climate Group, Oregon State University, http://prism.oregonstate.edu, created 8 Nov 2016). Yearly averages spanned 1996‐2013, which is the developmental period for the periodical cicada population that emerged in 2013. Because temperature measured at this scale may not capture acute effects of urbanization, we used latitude as our prediction factor (latitude was strongly correlated with temperature (Figure S2, *r*² = .91, *p* < .01)).

### 2.5 Statistical analyses

We used a Procrustes ANOVA in MorphoJ to test for measurement error and extract two measures of shape fluctuating asymmetry. The Procrustes distance is defined as the absolute measure of shape variation while the Mahalanobis distance is a transformation of the shape data so that there is equal variation in every direction (Klingenberg, 2015; Klingenberg & Monteiro, 2005).

We used a forward stepwise model selection approach to first determine the best model for our data, including predictor variables such as sex, latitude, habitat, state (to reflect citizen scientists’ sampling effort across range), and habitat–latitude interaction. Based on the minimum AICc criteria, we determined that all predictors, excluding state, provided the best model for our data (Table S1). We used a general linear model approach to assess body size as a function of habitat (rural vs. urban), sex, latitude, and habitat–latitude interaction. Because data for shape FA were not normally distributed, we used a generalized linear model (GLM) with a Poisson distribution to assess shape FA as a function of our predictors and their interaction. To account for any differences in development associated with sex, we analyzed shape and size for males and females independently (Leonard, 1964; White & Lloyd, 1975). Additionally, we ran a Wilcoxon rank sum test on urban and rural cicadas at the extreme ends of the range (36°N for southernmost range and 41°N for northernmost range) to compare body size and shape FA. All analyses were performed using JMP Pro 11.2.0 (SAS Institute, Cary, NC, USA).

## 3 RESULTS

### 3.1 Urbanization, latitude, and body size

In line with previous research (Koyama et al., 2015), we found that in rural areas the body size of cicadas was negatively associated with latitude, which is to say, southern cicadas were bigger and overall rural cicadas followed converse Bergmann's rule (Table 1; Figure 2b). However, this pattern was disrupted for cicadas collected in urban habitats (Figure 2b). In the northern part of the range (latitude 40°N and greater), urban cicadas were significantly larger than rural cicadas (Wilcoxon test, N_rural_ = 28, N_urban_ = 10 x^2^ = 6.0176, *p* = .01). However, rural and urban cicadas did not differ in body size in the south (latitude 36°N ‐ 37°N) (Wilcoxon test, N_rural_ = 26, N_urban_ = 34 x^2^ = 0.0080, *p* = .93).

**Table 1:** Results of general linear model analysis with habitat (rural vs urban), latitude, and interaction for wing size (Centroid size) in periodical cicadas (*N* = 163; 73 females and 90 males). Bold value indicate models that are statistical significance

Source	Estimate	*SE*	*t*	*p*
Whole model				
Intercept	830.68817	47.68551	17.42	**<.01**
Sex	13.671733	2.350126	5.82	**<.01**
Habitat	−2.578255	2.273218	−1.13	.26
Latitude	−4.411861	1.255794	−3.51	**<.01**
Habitat*Latitude	−3.494172	1.17189	−2.98	**<.01**
Female				
Intercept	942.1044	96.90706	9.72	**<.01**
Habitat	0.653786	3.535451	0.18	.85
Latitude	−7.05692	2.622771	−2.69	**.01**
Habitat*Latitude	−1.34508	2.622771	−0.51	.61
Male				
Intercept	789.6384	61.40458	12.86	**<.01**
Habitat	−4.06548	3.172457	−1.28	.20
Latitude	−3.70015	1.585738	−2.33	**.02**
Habitat*Latitude	−4.09716	1.585738	−2.58	**.01**

**Figure 3:**
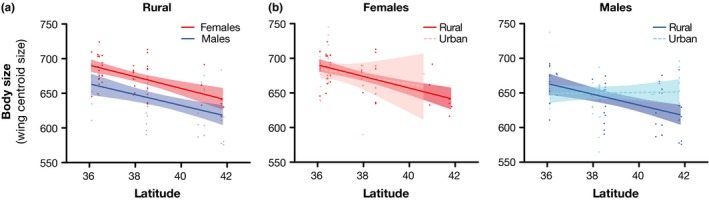
Body size for females and males in rural and urban habitats along Brood II's latitudinal range. Body size trends are disrupted in urban habitats for both sexes compared to rural populations (*p* = .02)

When comparing body size by sex, both males and females decreased in size with increasing latitude among rural sites (Table 1; Figure 3). The change in male body size across rural and urban habitats was statistically significant (Table 1; Figure 3). In the northern part of the range, male cicadas in urban habitats were 4% larger than those in rural habitats. We did not detect a significant difference in female body size between rural and urban habitats; however, few female cicadas were collected in urban habitats in the northern part of the range, which decreased our ability to detect differences in body size between rural and urban female cicadas in the north (Table 1; Figure 3).

### 3.2 Urbanization, latitude, and fluctuating asymmetry

Results from the Procrustes ANOVA indicated no significant effect of measurement error (Table 2). In addition to fluctuating asymmetry, where deviations from perfect symmetry occur randomly towards the left or right side, we detected the presence of directional asymmetry, which is a consistent bias in development toward only one side (Bookstein, 1991; Klingenberg & Monteiro, 2005; Table 2). Directional asymmetry is present in many organisms, including some insect species (Klingenberg, 2015), and we found evidence that cicada wings have a bias towards the left side. Thus, we used only transformed shape data (Mahalanobis distance) for all FA analyses as recommended by Klingenberg, 2015.

**Table 2:** Procrustes ANOVA of centroid size and shape of *Magicicada septendecim* forewings (*N* = 163) to assess the presence of measurement error, directional asymmetry (side), and fluctuating asymmetry (individual × side). Bold value indicate models that are statistical significance

	MS	*df*	*F*	*p*
Centroid size				
Individual	13980.7	27	9.95	**<.01**
Side	944968.7	1	672.39	**<.01**
Individual × side	1405.4	27	54.26	**<.01**
Measurement	25.9	56	0.03	1
Shape				
Individual	0.00017	1296	3.68	**<.01**
Side	0.014	48	304.82	**<.01**
Individual × side	0.000046	1296	6.01	**<.01**
Measurement	0.000007	2688	0.19	1

Overall, we found no effect of urbanization, latitude, or their interaction on levels of fluctuating asymmetry in *M. septendecim* wings (Table 3). Males and females analyzed independently also did not show a significant pattern in fluctuating asymmetry (Table 3), nor did comparisons of rural and urban cicadas at the extreme ends of the range (Southern: Wilcoxon test, *N*_rural_ = 26, *N*_urban_ = 34 x^2^ = 0.0376, *p* = .85; Northern: Wilcoxon test, *N*_rural_ = 28, *N*_urban_ = 10 x^2^ = 3.4462, *p* = .06).

**Table 3:** Results of GLM with habitat (rural vs urban), latitude, and interaction for wing shape FA (Mahalanobis) in periodical cicadas (*N* = 163; 73 females and 90 males)

Source	*df*	L‐R x²	*p*
Whole model			
Habitat	1	0.0173604	.90
Sex	1	0.0218162	.88
Latitude	1	0.3412416	.56
Habitat*Latitude	1	0.260124	.61
Female			
Habitat	1	0.007959	.93
Latitude	1	0.151667	.70
Habitat*Latitude	1	0.116893	.73
Male			
Habitat	1	0.062405	.80
Latitude	1	0.058798	.81
Habitat*Latitude	1	0.215907	.64

## 4 DISCUSSION

Urbanization is predicted to affect the physiological and metabolic condition of animal populations due to increased environmental stressors (e.g., disturbance, pollutants, etc.) as well as urban heating (the “urban heat island” effect). We found that urbanization disrupted a latitude–size relationship that is common to all periodical cicadas (Koyama et al., 2015). In rural populations, cicadas were larger at the southern end of their range compared with the northern end, which is consistent with the converse Bergmann's rule. In northern cities, however, urban cicadas were larger than rural cicadas and more similar in size to cicadas living to the south. A cicada living in an urban habitat in Poughkeepsie, NY, for example, was the same size as a cicada living 400 km to the south in rural Maryland. In contrast, cicadas living in cities in the southern end of their range did not differ in size from rural cicadas. Previous research has shown that introduction of invasive species (Blanchet et al., 2010) and changes in diet (Diamond & Kingsolver, 2010) can disrupt latitude–body size relationships in animals, and our results show that urbanization may be an additional disruptor.

Urban warming in the northern sites has created thermal conditions similar to those in southern rural sites, which could have allowed northern cicadas to achieve as much growth in cities as they would in rural environments to the south. In the daytime, temperatures in the city can be up to 8°C warmer than temperatures in rural habitats, and the effects of urban heating are particularly strong in cities in the eastern United States (Imhoff et al., 2010).). Cicadas that are pushed closer to their thermal optimum may therefore increase in body size (Karban, 1983, 1997; Lloyd & Dybas, 1966). The fitness consequences for the population remain to be explored. On the one hand, larger body size in urban cicadas may lead to increased fecundity in females and more attractive mating calls in males (Angilletta, Steury, & Sears, 2004; Brown & Chippendale, 1973). Alternatively, cicadas pushed away from an evolved physiological optimum may experience a reduction in fitness if, for instance, urban warming disrupts the timing of emergence and the availability of mates between urban and rural populations.

Periodical cicadas may be sensitive to a converse Bergmann's rule effect because they feed primarily on xylem fluid in tree roots, which consists of water and inorganic ions, and consequently grow very slowly (White & Strehl, 1978). Urbanization, whether through warming, landscape fragmentation, or pollution, may affect xylem quality and availability. Urban trees tend to have higher incidences of xylem cavitation due to urban warming (Bush et al., 2008; Litvak, McCarthy, & Pataki, 2012; Savi, Bertuzzi, Branca, Tretiach, & Nardini, 2015) and are of poorer quality compared to rural trees (McDonnell et al., 1997). Conversely, urban horticultural practices, such as the use of fertilizer and water supplementation, may have a positive impact on the quality of xylem fluid as cicadas associated with fertilized trees develop faster, have larger body sizes, and are found typically in higher densities (Karban, 2014; White & Lloyd, 1985; White, Lloyd, & Zar, 1979). The degree to which urban resource quality impacts cicada fitness along a latitudinal gradient remains unclear but given that early cicada growth appears to be sensitive to food availability and quality (White & Lloyd, 1975) one might expect these conditions to impose a differential selective pressure on body size if, for example, resources are more readily available in the south compared to northern end of the range.

We found no significant effect of urbanization on fluctuating asymmetry—a measure of developmental stress—in *M. septendecim* despite their exceptionally long lifespan and long‐term exposure to potential stressors. Our findings are similar to studies that have investigated insects in rural–urban habitats and found no significant change in fluctuating asymmetry (Elek, Lövei, & Bátki, 2014; Weller & Ganzhorn, 2004). One limitation of assessing developmental stress in the field is measuring the degree of selection against developmentally unstable individuals (Møller, 1997). More asymmetrical individuals may be removed from the population prior to sampling and thus stressed populations may appear more symmetrical (and larger). We also detected directional asymmetry in our populations, which would also affect our ability to detect differences in fluctuating asymmetry due to environmental stressors. While there is evidence that directional asymmetry and fluctuating asymmetry are often interrelated (Graham, Emlen, Freeman, Leamy, & Kieser, 1998; Lens et al., 2000), it remains unclear from our study whether or not *M. septendecim* is experiencing developmental stress in urban habitats based on results from fluctuating asymmetry alone. Therefore, we cannot completely rule out the possibility that urbanization negatively impacts cicada development.

The disruption of the latitude–size relationship in urban cicadas raises evolutionary questions about how the expansion of urban areas will affect cicada populations. A key feature of a population's ability to respond to environmental change is the degree of developmental plasticity in the population (Sgrò, Terblanche, & Hoffmann, 2016; West‐Eberhard, 2005). Periodical cicadas exhibit some degree of plasticity in life cycle development as indicated by incidences of nonsynchronous emergences due to changes in environmental cues (Marshall, Cooley, & Hill, 2011). Our finding of changes in body size across the rural–urban gradient adds support to the possible role of plasticity in cicada evolution. Urbanization may constrain or speed up the population's adaptive response to the environmental changes, and this is particularly relevant given current predicted changes associated with climate change.

There is strong public concern for the status of periodical cicadas, which is exemplified by the citizen scientists who contributed to this project as well as a host of other citizen science projects that focus on periodical cicadas (Beasley, Benson, Welch, Reid, & Mousseau, 2012; Kritsky, 1992). Periodical cicadas only occur in the eastern part of North America, an area that is already the most urbanized of the United States and is likely to become much more urbanized in the next decades (Terando et al., 2014). Two periodical cicada broods have gone extinct within the last 150 years, one of which—the Floridian brood, XXI—was distributed farthest to the south (Young, 1958). We propose that continued monitoring of periodical cicadas in urban habitats, including a more fine scale assessment of habitat conditions, is needed to understand how urbanization could affect cicadas over longer time scales and in earlier development stages.

In conclusion, our study further illustrates the implications of increasing urbanization on the cicada's evolutionary trajectory and how the degree and direction of those impacts may vary depending on a species geographic range.

## CONFLICT OF INTERESTS

We have no competing interests.

## DATA ACCESSIBILITY

The dataset supporting this article is available from FigShare: https://figshare.com/s/6be12546629009d89545.

## AUTHORS’ CONTRIBUTIONS

DEB, CAP, and RRD analyzed the data and wrote the manuscript, NB collected the geometric morphometric data, and HLM coordinated citizen scientists and collected samples. All authors gave final approval before publication.
