# Supplementary figures and images for: Urbanization disrupts latitude‐size rule in 17‐year cicadas
Source: Ecol Evol. 2018 Feb 2;8(5):2534–41. doi: 10.1002/ece3.3879 (PMC5838052; doi:10.1002/ece3.3879)

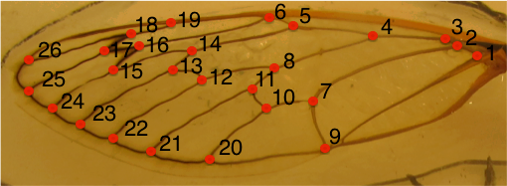

Supplement: Supplementary file 1 [file ECE3-8-2534-s001.png]
