# Supplementary material for: Urbanization disrupts latitude‐size rule in 17‐year cicadas
Source: Ecol Evol. 2018 Feb 2;8(5):2534–41. doi: 10.1002/ece3.3879 (PMC5838052; doi:10.1002/ece3.3879)
Supplement: Supplementary file 3 [file ECE3-8-2534-s003.docx]

Table S1: Results of forward stepwise model selection approach using a minimum AICc criteria. A predictor variable is added to the model with each step. Best model is indicated by the smallest AICc value.

| **Parameter** | **SeqSS** | **RSquare** | **Cp** | **p** | **AICc** |
| --- | --- | --- | --- | --- | --- |
| Sex | 43236.92 | 0.2288 | 31.811 | 2 | 1576.45 |
| Latitude | 18515.38 | 0.3268 | 9.5697 | 3 | 1556.4 |
| Habitat*Latitude | 6745.907 | 0.3625 | 4.7375 | 5 | 1551.81 |
| State | 671.8455 | 0.3776 | 9 | 9 | 1556.81 |
